# Host‐Associated School Integration and Behaviour Among Juvenile *Caranx crysos* and *Sarpa salpa* in a Shallow Mediterranean Habitat

**DOI:** 10.1002/ece3.74583

**Published:** 2026-10-22

**Authors:** V. Basile, E. Brunelli, M. Mezzasalma

**Affiliations:** ^1^ Department of Biology, Ecology and Earth Sciences (DiBEST) University of Calabria Cosenza Italy

**Keywords:** behaviour, fish, interspecific associations, Mediterranean, school

## Abstract

Interspecific associations between fishes can generate complex behavioural interactions, yet their occurrence and functional significance in temperate marine systems remain poorly documented. Here we describe a previously unreported case of host‐associated behaviour involving juvenile *Caranx crysos* and schools of juvenile *Sarpa salpa* in coastal Mediterranean habitats. Associations were recorded exclusively in *C. crysos* individuals ≤ 15 cm TL, suggesting a size‐ and context‐dependent pattern of association with the host schools. Juvenile *C. crysos* integrated into *S. salpa* schools by adopting spatial positioning and behavioural patterns consistent with social mimicry. This association may provide antipredator advantages through group integration, while also allowing opportunistic foraging on benthic organisms and resuspended organic particles generated during host grazing activity. In rare cases, multiple individuals of *C. crysos* co‐occurred within the same school, producing aggressive interactions and indicating intraspecific competition for access to the host aggregation. From the host perspective, *S. salpa* exhibited only brief and limited behavioural responses, with no evidence of persistent exclusion or disruption of schooling behaviour, suggesting a low‐cost, largely tolerated interaction. Overall, our observations suggest the occurrence of a host‐related behavioural strategy in juvenile *C. crysos* that combines group integration, commensalistic and opportunistic feeding and intraspecific competition, likely shaped by ontogenetic constraints. These results highlight a previously overlooked level of behavioural complexity in temperate fish assemblages and suggest that fine‐scale interspecific interactions in Mediterranean coastal ecosystems may be more widespread than currently recognised.

## Graphical abstract

In this work we report a case of interspecific aggregation between *Caranx crysos* and *Sarpa salpa* in coastal Tyrrhenian waters. Associations were observed only in juvenile *C. crysos* , indicating a size‐dependent relationship with *S. salpa* schools. Juveniles integrated into host schools through social mimicry‐like behaviour, likely gaining antipredator benefits and feeding opportunistically on benthic prey and particles resuspended during host grazing. Occasional aggressive interactions among conspecifics suggested competition for access to host groups. *S. salpa* showed minimal behavioural responses, indicating a low‐cost and largely tolerated interaction. Overall, these observations point to an ontogenetically driven behavioural strategy in juvenile *C. crysos* combining group integration, commensal feeding and intraspecific competition.

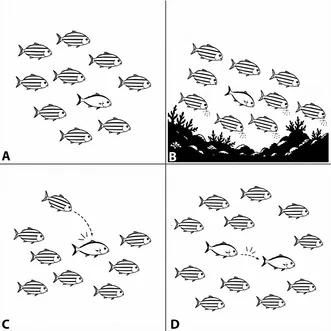

## 1 Introduction

Mixed‐species groups (MSGs) comprise individuals belonging to two or more species that form sufficiently close associations to be regarded as members of a single social group (Goodale et al. 2017). Such interspecific aggregations have been documented across numerous taxa and ecological contexts and represent a widespread ecological phenomenon in aquatic environments (Shaw 1978). Based on the duration of association, heterospecific schools can be broadly distinguished into stable and opportunistic mixed‐species groups, while their functional role is commonly classified as antipredatory, foraging or habitat‐driven (Paijmans et al. 2019, 2021; Mukherjee and Bhat 2025). With respect to species composition and relative abundance, mixed‐species schools may be further described as dominant (when one species is more numerous and behaviourally influential), co‐dominant (when two or more species are present in similar numbers and exert comparable influence) or heterosingleton associations (when a single individual of one species joins a school of a different species) (Tang et al. 2017; Webster et al. 2025).

During early developmental stages, individuals are more likely to form MSGs than adults, due to increased vulnerability to predation and the need to reduce energetic costs associated with foraging and risk assessment (Parrish and Edelstein‐Keshet 1999; Haak et al. 2020). A key mechanism underlying these associations is the use of social information provided by host species, which are capable of rapidly detecting and responding to environmental or predatory cues. Association with such species may enhance collective vigilance and improve access to food resources, particularly when host feeding activities, such as substrate disturbance, increase prey availability in the water column (Srinivasan et al. 2010; Haak et al. 2020).

Potential costs of interspecific aggregations include increased competition and the oddity effect, whereby individuals that differ morphologically or behaviourally from the group experience elevated predation risk (Ward et al. 2002). To mitigate these costs, many fish species preferentially associate with morphologically similar individuals, thereby enhancing group cohesion and reducing predator targeting (Penry‐Williams et al. 2018). Such morphological convergence is particularly common in juveniles, which often share early developmental traits and occupy shallow coastal habitats that function as nursery areas (Cheminée et al. 2021). Mixed‐species associations may also involve behavioural mimicry, whereby individuals replicate the movement patterns or trajectories of another species without necessarily sharing its trophic niche (Dafni and Diamant 1984; Randall and McCosker 1993). This strategy is well documented in coral reef fishes during juvenile stages and may provide both antipredator and foraging benefits (Haak et al. 2020). However, similar examples are currently poorly explored in temperate marine ecosystems such as the Mediterranean Sea.

In the Mediterranean, *Sarpa salpa* is a gregarious herbivorous species characterised by selective grazing behaviour in *Posidonia oceanica* seagrass meadows. Juvenile individuals form cohesive yet flexible schools that balance predator avoidance and foraging efficiency and may function as transient microhabitats that attract other species (Buñuel et al. 2020; McGonagle et al. 2023). In contrast, *Caranx crysos* is a coastal‐pelagic opportunistic predator whose juveniles are frequently associated with structurally complex habitats, such as artificial reefs and oil platforms, which provide refuge and feeding opportunities (Brown et al. 2010). Dietary studies further indicate an ontogenetic shift from crustacean‐dominated diets in small individuals to more piscivorous strategies in larger ones, highlighting the species' trophic flexibility (Sley et al. 2009).

Associations between juvenile carangids and other organisms have been reported in different regions. For instance, juveniles of *Caranx caballus* , *Chloroscombrus orqueta* and *Hemicaranx leucurus* have been observed associating with the scyphozoan *Stomolophus meleagris* in the Gulf of California, likely as a protective strategy (Reza et al. 2018). However, to date, no association between *C. crysos* and *S. salpa* has been documented in either tropical or temperate systems.

Documenting novel interspecific behaviours is crucial not only for understanding species‐specific adaptive strategies, but also for revealing the functional complexity of marine ecosystems such as those in the Mediterranean. In this context, the present study reports an undocumented host‐associated behaviour involving juvenile *C. crysos* and schools of *S. salpa* in a coastal area of the southern Tyrrhenian Sea. Specifically, we describe the occurrence, frequency and behavioural features of this association, and discuss its potential functional significance in terms of mixed‐species shoaling, opportunistic foraging and ontogenetic habitat use.

## 2 Materials and Methods

Underwater observations were conducted while snorkelling in shallow coastal waters between July and August 2025 using visual census methods. The study area was located along a coastline of approximately 2 km between Case del Conte and Ogliastro Marina (southern Tyrrhenian Sea), within a heterogeneous environment characterised by *Posidonia oceanica* patches and mixed rocky–sandy substrates, near the Marine Protected Area of Santa Maria di Castellabate (Campania, Italy).

During each survey, two observers independently conducted 30‐min roving transects (De Girolamo and Mazzoldi 2001) at depths of 0.5–2 m from the shoreline, following consistent directions to minimise the probability of repeatedly encountering the same fish schools. A total of 20 h of transect observation across 40 transects was completed (10 h and 20 transects per observer). These 20 h of observations were distributed over 5 days, with four 30‐min transects conducted in the morning and four 30‐min transects in the afternoon‐evening on each day (4 h of observation per day).

At the start of each transect, observers activated a 30‐min timer and recorded data on underwater PVC slates, including: (i) the number of *Sarpa salpa* schools encountered; (ii) the predominant size class of individuals within each school (≤ 15 cm TL and > 15 cm TL); (iii) school abundance class (i.e., number of individuals per school ≤ 15, 16–30, 31–45 and > 45 individuals); (iv) the presence and number of associated *Caranx crysos* individuals and their size class; and (v) the observed behaviours. When a heterospecific association was identified, the 30‐min transect timer was stopped and the association was followed for 10 min, during which the behaviour and interactions of the associated *C. crysos* and *S. salpa* individuals were continuously monitored, recorded and photographically documented. The transect timer was then resumed. Differences in the occurrence of *C. crysos* associations among *S. salpa* school size and abundance classes were assessed using Fisher's exact tests (Fisher 1922) due to low expected frequencies and the presence of zero observations in one size category (see Results). Variation in recorded heterospecific associations was assessed using a chi‐square goodness‐of‐fit test to compare observed frequencies among the five sampling days, assuming equal expected frequencies given the identical sampling effort among days. Differences between morning and afternoon‐evening transects in the frequency of recorded associations were tested using a two‐tailed exact binomial test with an expected probability of 0.5 for each period. Significance was set at α = 0.05. All remaining quantitative data are presented as descriptive statistics, as the primary aim of the study was to characterise behavioural interactions rather than test predictive models.

## 3 Results

In total, we observed *n* = 195 schools of *S. salpa* . Associations between *C. crysos* and *S. salpa* (Figure 1, Supporting Information, Figure S1–S5) differed significantly between host school size classes (Fisher's exact test, *p* = 0.037) (Figure 2). Associations were recorded in 14 of the 195 *S. salpa* schools observed (7.2%). In particular, associations occurred in 14 of 154 schools (9.1%) composed of individuals ≤ 15 cm TL, whereas no associations were observed in schools belonging to larger size classes (> 15 cm TL; *n* = 41). Schools composed of individuals > 15 cm TL were therefore excluded from subsequent quantitative analyses. All associated *C. crysos* individuals were in the same size class as the *S. salpa* individuals composing the host school.

**FIGURE 1:**
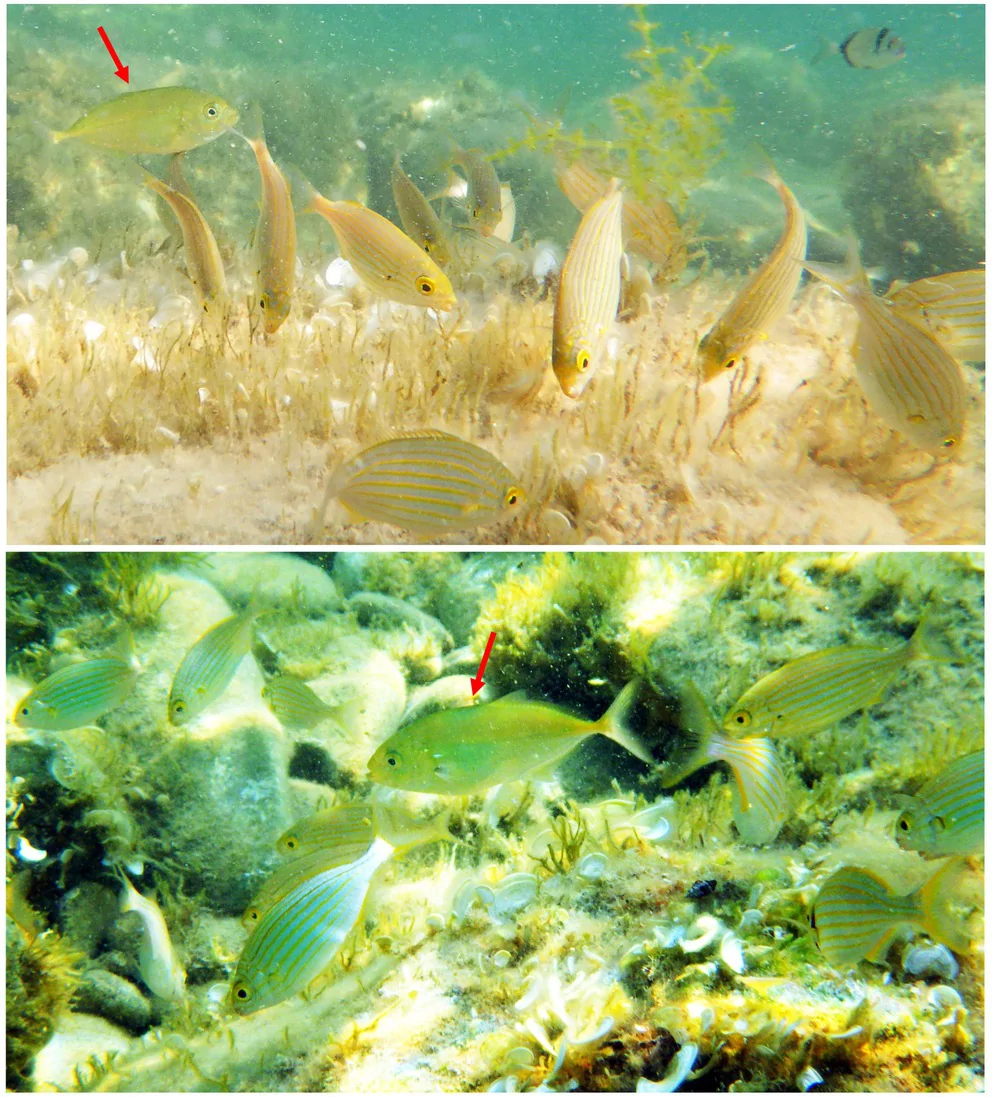
Association of juvenile *C. crysos* to two different schools of *S. salpa* . Red arrows point at *C. crysos* .

**FIGURE 2:**
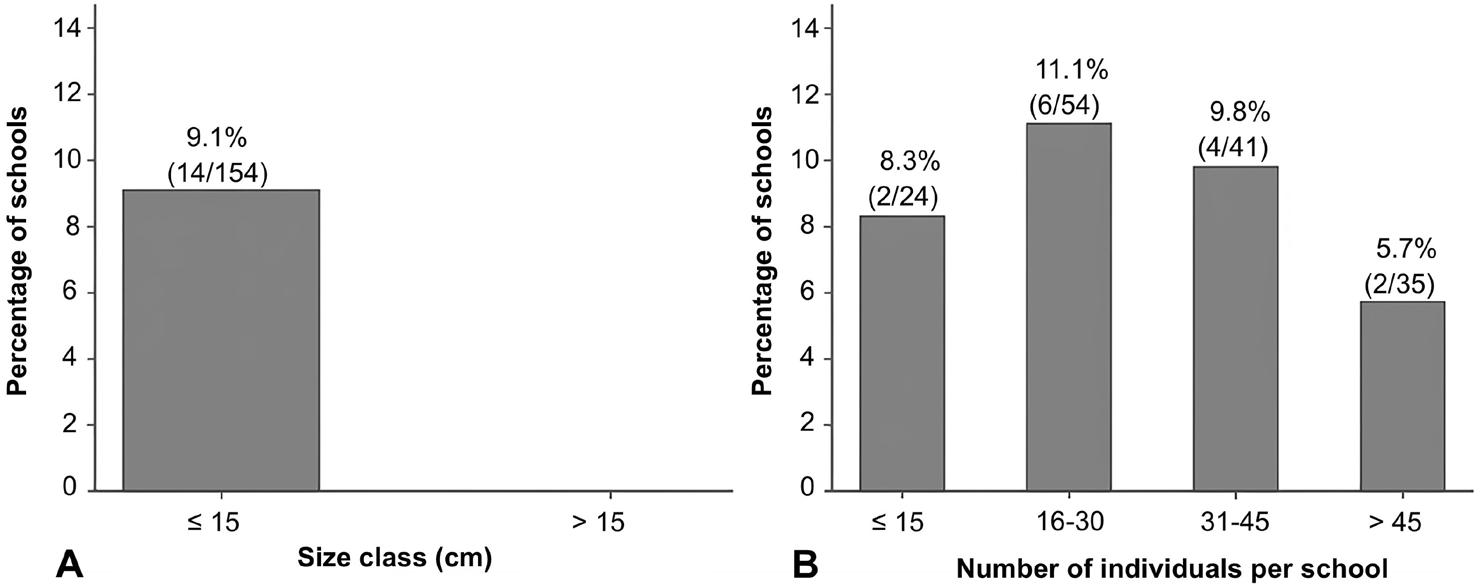
Results of the Fisher's exact tests showing association frequency between *C. crysos* and *S. salpa* by (A) size class (*p* = 0.037*) and (B) by numbers of individuals per school (*p* = 0.05). Absolute numbers are reported in parentheses.

Among the 14 associated schools, a single *C. crysos* individual was observed in 11 cases, two individuals in two cases and four individuals in one case. Overall, 19 *C. crysos* individuals were recorded in association with *S. salpa* schools, corresponding to a mean of 1.36 ± 0.84 SD individuals per associated school (range = 1–4).

The occurrence of *C. crysos* associations did not differ among school abundance classes (Fisher's exact test, *p* > 0.05) (Figure 2). Associations were observed in 2 of 24 schools (8.3%) in the ≤ 15‐individual class, 6 of 54 schools (11.1%) in the 16–30‐individual class, 4 of 41 schools (9.8%) in the 31–45‐individual class and 2 of 35 schools (5.7%) in the > 45‐individual class. Numbers of recorded heterospecific associations had no significant variation among sampling days (2–4 per day, χ^2^ = 1.00, df = 4, *p* = 0.910) or between morning and afternoon‐evening periods (6 and 8, respectively; exact binomial test, *p* = 0.791).

During surveys, *C. crysos* individuals were never observed alone, forming conspecific schools or associating with other species.

When associations between *C. crysos* and *S. salpa* schools were observed, the following behaviours were consistently recorded (Figure 3): (i) integration of *C. crysos* individuals into *S. salpa* schools, with swimming speed, trajectories and inter‐individual distances similar to those of host school members (Figure 3A); (ii) opportunistic foraging, whereby juvenile *C. crysos* remained in close proximity to feeding *S. salpa* individuals, exploiting small benthic organisms and resuspended organic particles generated by the grazing activity of the host species (Figure 3B); (iii) host responses, in which *S. salpa* individuals exhibited brief chasing behaviour towards *C. crysos* individuals, although carangids typically rejoined the school immediately thereafter (Figure 3C); and (iv) intraspecific aggression, observed when more than one *C. crysos* individual was present within the same school, including brief aggressive turns directed towards conspecifics, although no subsequent chasing or displacement was observed (Figure 3D).

**FIGURE 3:**
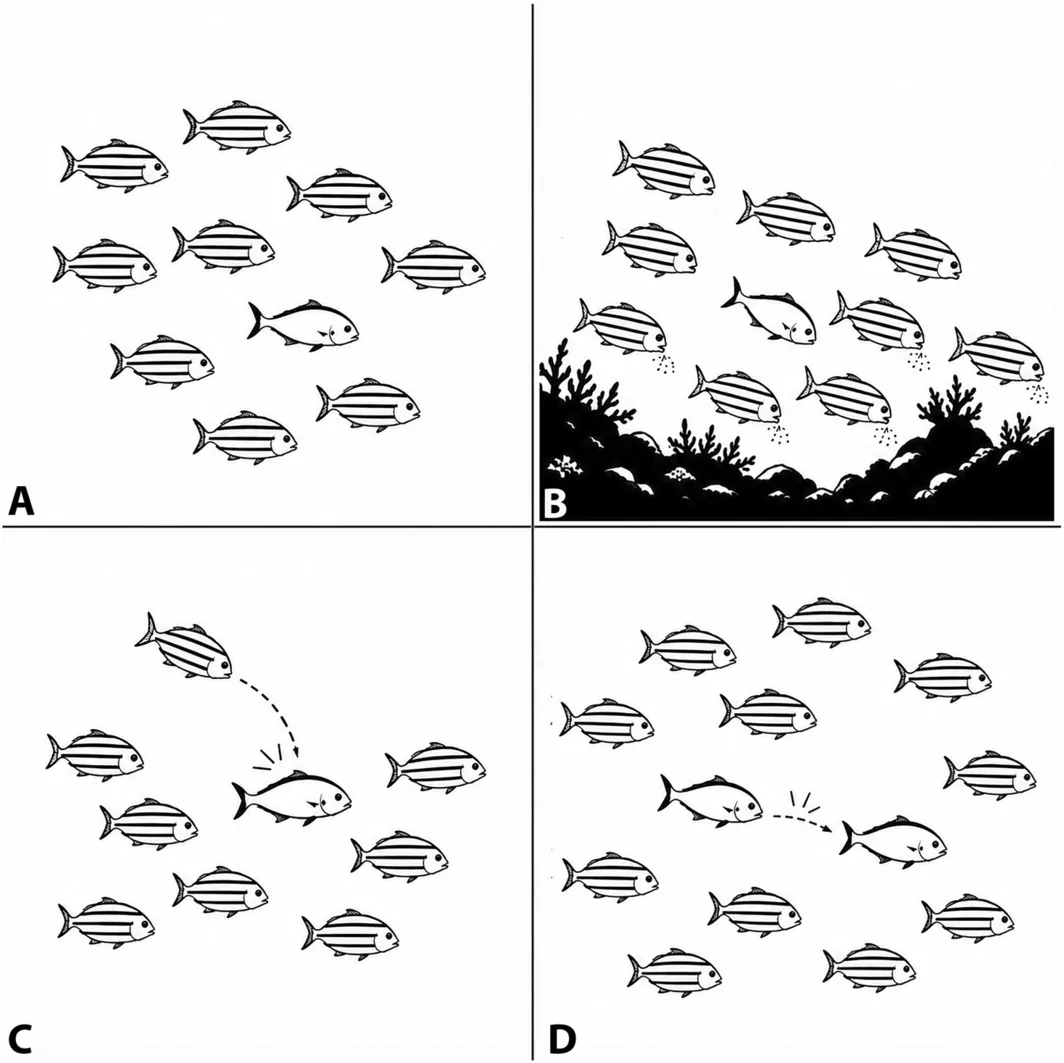
(A) Association of *C. crysos* with *S. salpa* schools, involving integration into the school and maintenance of inter‐individual distances; (B) opportunistic foraging, by *C. crysos* , exploiting small organisms and organic particles made available by host grazing activity (C) responses of *S. salpa* individuals, exhibiting brief chasing behaviour towards *C. crysos* individuals; (D) aggressive behaviour among *C. crysos* individuals within the same school of *S. salpa* school.

The behaviours described above did not follow a fixed sequence, and their temporal order varied among observations. No instances of *C. crysos* detaching from *S. salpa* schools were observed. Excluding the interspecific interactions described above, no obvious differences were noted in the behaviour of *S. salpa* individuals in heterospecific schools with *C. crysos* compared with that typically observed in monospecific *S. salpa* schools, including general swimming speed, feeding activity, turning behaviour, and school cohesion.

## 4 Discussion

The observations reported in this study document a previously undescribed interspecific association between juvenile *C. crysos* and schools of juvenile *S. salpa* . Our results suggest that this association may be size‐ and context‐dependent, as *C. crysos* was recorded only with schools composed of small *S. salpa* individuals. The observed behavioural repertoire further suggests that juvenile *C. crysos* may derive multiple potential benefits from this interaction. A first possible advantage is reduced predation risk, achieved through mixing into a heterospecific school and adopting a behaviour similar to that of individuals of the host species (see Pereira et al. 2011). By integrating into *S. salpa* shoals and maintaining inter‐individual spacing similar to that of school members, juvenile *C. crysos* may benefit from the antipredator advantages typically associated with group living, including dilution, confusion effects and increased vigilance (Foster and Treherne 1981; Krakauer 1995; Boland 2003). Similarity in body size, coloration and swimming behaviour between juvenile *C. crysos* and *S. salpa* likely represents a key prerequisite for the formation of mixed‐species shoals (Srinivasan et al. 2010). Such similarity may promote group cohesion, synchronised movements and antipredator benefits (Landeau and Terborgh 1986; Peuhkuri 1997), while simultaneously reducing the potential costs associated with the oddity effect (Rodgers et al. 2011).

A second potential advantage arises from feeding opportunities associated with the grazing activity of *S. salpa* . By remaining in close proximity to feeding individuals, carangids may exploit small benthic organisms and organic particles resuspended into the water column, thereby gaining access to trophic resources that would otherwise be unavailable or more energetically costly to obtain independently.

The low frequency of the observed interspecific association can be attributed to the relative rarity of *C. crysos* in comparison to the host species. Additionally, association with *S. salpa* schools may decrease the costs related to the formation of conspecific shoals among carangids, particularly by mitigating heightened foraging competition (Grand and Dill 1999). Moreover, in the rare instances where multiple *C. crysos* individuals were present within the same *S. salpa* school, active aggressive interactions were observed, suggesting that access to the host shoal is a competitively contested and valuable resource for carangids. Similar patterns have been reported in juvenile parrotfish, where spatial competition within mixed‐species groups is associated with increased aggression among conspecifics (Overholtzer and Motta 2000).

Concerning the host species, *S. salpa* showed only limited behavioural responses to the presence of *C. crysos* , consisting of brief chasing events after which individuals of the associated species rapidly rejoined the school. No persistent exclusion of carangids from the schools was observed, suggesting that the interaction is largely tolerated rather than actively avoided. Overall, no clear benefit to the host species was detected, while any associated costs appear to be low and transient, and insufficient to disrupt school cohesion or foraging behaviour. These findings suggest that juvenile carangids were not perceived as a significant disruptive presence by *S. salpa* , resulting in a predominantly low‐impact interaction from the host's perspective. This observation aligns with previous reports of tolerated host–commensal relationships in other fish species (Ochi and Yanagisawa 2005).

The complex interaction documented here likely represents an ontogeny‐driven strategy in *C. crysos* , involving a combination of host‐specific behavioural mimicry and commensal‐like use of heterospecific schools. This interpretation is consistent with recent perspectives in fish ecology emphasising behavioural flexibility as a key adaptation to structurally complex and heterogeneous habitats, such as shallow rocky substrates and seagrass meadows (Brodeur et al. 2017; Xing et al. 2025). In this context, the behavioural plasticity exhibited by *C. crysos* may constitute a context‐dependent adaptive strategy that provides selective advantages during early developmental stages, particularly in nursery environments where refuge availability and trophic conditions are highly variable. Comparable levels of behavioural complexity have been widely reported in juvenile fishes from tropical systems, especially in mixed‐species shoaling, commensal interactions and behavioural mimicry (Haak et al. 2020). However, analogous interactions appear to be far less documented in temperate marine habitats and remain relatively underreported in the Mediterranean Sea.

We also note that the observed behaviours, together with the overall phenotypic similarity between juvenile *C. crysos* and *S. salpa* , share some characteristics with the school‐oriented mimicry described by Dafni and Diamant (1984), later referred to as social mimicry by Randall and McCosker (1993). However, unlike some documented examples of social mimicry, juvenile *C. crysos* do not closely match the fine‐scale colour patterns of *S. salpa* . Rather, the resemblance is based on a broader similarity in overall appearance, including body shape, coloration and size, which may reduce visual discrimination between the two species when individuals are integrated within heterospecific schools.

It should also be noted that young individuals of *C. crysos* (at least up to 14 cm) have been reported to display a more yellowish coloration than bigger individuals (Psomadakis et al. 2011), thus potentially making them more visually similar to *S. salpa* and facilitating mixed‐species grouping at younger stages.

Altogether, this contribution highlights the adaptive complexity of juvenile fish behaviour in temperate ecosystems and suggests that such interactions may represent transient ontogenetic strategies that are more widespread than currently recognised, yet overlooked in fine‐scale behavioural studies of Mediterranean fish communities. Future studies employing quantitative analyses could better define the mechanisms underlying the association between *C. crysos* and *S. salpa* by disentangling the relative roles of visual similarity, social behaviour, predation risk and foraging opportunities.

## Author Contributions

**V. Basile:** data curation (equal), formal analysis (equal), investigation (equal), methodology (equal), writing – original draft (lead), writing – review and editing (equal). **E. Brunelli:** data curation (equal), formal analysis (equal), investigation (equal), methodology (supporting). **M. Mezzasalma:** conceptualization (lead), data curation (equal), formal analysis (equal), investigation (lead), methodology (equal).

## Funding

The authors have nothing to report.

## Conflicts of Interest

The authors declare no conflicts of interest.

## Supporting information


**Figure S1:** Recorded case of interspecific association between *C. crysos* and *S. salpa* . The red arrow points at *C. crysos* .
**Figure S2:** Recorded case of interspecific association between *C. crysos* and *S. salpa* . The red arrow points at *C. crysos* .
**Figure S3:** Recorded case of interspecific association between *C. crysos* and *S. salpa* . The red arrow points at *C. crysos* .
**Figure S4:** Recorded case of interspecific association between *C. crysos* and *S. salpa* . The red arrow points at *C. crysos* .
**Figure S5:** Recorded case of interspecific association between *C. crysos* and *S. salpa* . Red arrows indicate *C. crysos* individuals.

## Data Availability

Data supporting the findings of this study are available within the manuscript and in the Supporting Information (Figures S1–S5).
