# Supplementary material for: Host‐Associated School Integration and Behaviour Among Juvenile Caranx crysos and Sarpa salpa in a Shallow Mediterranean Habitat
Source: Ecol Evol. 2026 Oct 22;16(10):e74583. doi: 10.1002/ece3.74583 (PMC13691172; doi:10.1002/ece3.74583)
Supplement: Supplementary file 1 [file ECE3-16-e74583-s001.odt]

Host-associated school integration and behaviour among juvenile Caranx crysos and Sarpa salpa in a shallow Mediterranean habitat
Basile V., Brunelli E., Mezzasalma M

Figure S1. Recorded case of interspecific association between C. crysos and S. salpa. The red arrow points at C. crysos.Figure S2. Recorded case of interspecific association between C. crysos and S. salpa. The red arrow points at C. crysos.


Figure S3. Recorded case of interspecific association between C. crysos and S. salpa. The red arrow points at C. crysos.

Figure S4. Recorded case of interspecific association between C. crysos and S. salpa. The red arrow points at C. crysos.


Figure S5. Recorded case of interspecific association between C. crysos and S. salpa. Red arrows indicate C. crysos individuals.
